# Analysis of Codon Usage Bias in the *Streptococcus pneumoniae* Pneumolysin Gene

**DOI:** 10.1155/cjid/2707639

**Published:** 2026-04-05

**Authors:** Xiaochun Tan, Hui Zhou, Jian Jiang, Yu Lu, Junyan Lu, Weifeng Shen

**Affiliations:** ^1^ Department of Laboratory Medicine, The First Hospital of Jiaxing, Affiliated Hospital of Jiaxing University, Jiaxing, China, jxdyyy.com

**Keywords:** codon usage bias, CpG dinucleotides, host adaptation, immune evasion, natural selection, pneumolysin, *Streptococcus pneumoniae*

## Abstract

*Streptococcus pneumoniae* pneumolysin is a key virulence factor belonging to the cholesterol‐dependent cytolysin family, enabling host cell lysis and immune evasion. While synonymous codon usage bias is known to fine‐tune virulence gene expression in pathogens, its role in pneumolysin remains uncharacterized. This study presents a comprehensive analysis of codon usage patterns in the pneumolysin gene across 420 curated coding sequences. We found a pronounced preference for A/U‐ending codons, significant underrepresentation of CpG dinucleotides, and moderate overall bias (effective number of codons, ENC = 50.28). Neutrality plot, parity rule 2 (PR2) bias, and ENC‐plot analyses collectively indicated that natural selection—not mutational pressure—is the dominant evolutionary force shaping this bias. Strikingly, pneumolysin’s codon usage showed a significant correlation with the abundant tRNA gene pool of its human host, suggesting an adaptive strategy that may minimize immunostimulation caused by bacterial mRNA release during infection. These findings reveal a balance between translational efficiency, proper protein folding, and immune evasion, thereby providing a functional understanding of pneumolysin evolution and a foundation for practical applications. These include guiding codon‐optimized heterologous expression for biochemical studies and enabling codon deoptimization for the design of safer live‐attenuated vaccines.

## 1. Introduction

*Streptococcus pneumoniae* is a major human bacterial pathogen responsible for significant global morbidity and mortality, causing diseases, such as pneumonia, meningitis, and sepsis, particularly in young children, the elderly, and immunocompromised individuals. A key determinant of its virulence is pneumolysin, a 53‐kDa pore‐forming exotoxin and a critical member of the cholesterol‐dependent cytolysin (CDC) family [1]. Released primarily upon bacterial autolysis, this thiol‐activated toxin binds specifically to membrane cholesterol in eukaryotic cells, oligomerizing to form transmembrane pores that cause direct cytolysis through osmotic disruption [2, 3]. Beyond pore formation, pneumolysin exhibits significant pro‐inflammatory activities: It activates the classical complement pathway by binding immunoglobulin G (IgG), inhibits neutrophil phagocytosis and respiratory burst, and drives tissue damage, inflammation, and immune evasion [4]. Consequently, pneumolysin facilitates bacterial colonization, dissemination across host barriers, and severe complications in pneumococcal diseases, making it a prime target for therapeutics, such as neutralizing antibodies, small‐molecule inhibitors, and next‐generation vaccines [5, 6].

The genetic code provides the fundamental blueprint for protein synthesis, translating nucleotide triplets (codons) into specific amino acids. While multiple synonymous codons can specify the same amino acid, organisms universally exhibit codon usage bias—a consistent, nonrandom preference for certain synonymous codons over others within their genomes [7]. This bias is far from a neutral evolutionary artifact; it serves as a critical regulatory mechanism with profound functional consequences. Specifically, codon preference directly influences translation efficiency (including both the speed and accuracy of the ribosomal machinery) and governs protein folding kinetics by modulating the rate of translation elongation, thereby shaping cotranslational folding pathways [8, 9]. Collectively, these effects significantly impact protein expression levels, protein function, and ultimately, cellular fitness. Consequently, in pathogenic bacteria, the strategic optimization of codon usage within virulence‐associated genes (such as those encoding toxins, adhesins, or secretion systems) represents a key adaptive strategy. By fine‐tuning the expression and functionality of these critical virulence factors, codon bias enhances the pathogen’s ability to colonize host tissues, evade immune responses, and cause disease [8, 10].

Despite extensive characterization of pneumolysin’s biochemical and immunological functions, the role of synonymous codon usage in its expression, regulation, and evolution remains largely unexplored. This study aims to fill this gap by systematically analyzing the codon usage patterns in the pneumolysin gene across a broad dataset of *S. pneumoniae* isolates. Our specific objectives are as follows: (1) to quantify the extent and pattern of codon usage bias in the pneumolysin gene; (2) to determine the relative contributions of mutational pressure and natural selection in driving this bias; and (3) to assess whether the codon usage aligns with the host tRNA pool, which would suggest an evolutionary adaptation for efficient translation or immunomodulation. By linking codon usage to pneumolysin’s virulence and host adaptation, this work provides novel evolutionary insights and a practical foundation for improving pneumolysin‐based antigen production and designing codon‐deoptimized live‐attenuated vaccines.

## 2. Methods

### 2.1. Data Collection

Pneumolysin gene sequences were retrieved from the NCBI GenBank database. To maintain genomic integrity, stringent quality criteria were implemented: (1) coding sequence (CDS) lengths between 1300 and 1700 bp, with divisibility by three to maintain complete codon alignment; and (2) all CDSs necessitated initiation by an ATG start codon and termination by canonical stop codons (TAA, TAG, or TGA), with absence of internal stop signals. After filtering, 420 sequences were included in this study. The corresponding accession numbers are provided in Supporting Table S1.

### 2.2. Nucleotide Composition Analysis

Nucleotide compositional analysis of pneumolysin CDSs was performed focusing specifically on third‐position bases within synonymous codons (%A3s, %C3s, %T3s, and %G3s), utilizing CodonW software (Version 1.4.2). Concurrently, position‐specific G + C content (GC1, GC2, and GC3) was calculated across all codon positions using EMBOSS Explorer’s bioinformatic toolkit (https://www.bioinformatics.nl/emboss-explorer/).

### 2.3. Relative Synonymous Codon Usage (RSCU) Analysis

The RSCU index was calculated for the pneumolysin gene to assess codon usage bias. This index quantifies the deviation of codon usage from equal preference by comparing the observed frequency of each codon to its expected frequency, assuming all synonymous codons for a given amino acid are used equally [11], and is computed using the formula:

(1) RSCU=Xij∑jniXijni

(where *X*_*i* *j*_ = frequency of *j*_*t* *h*_ codon for *i*_*t* *h*_ amino acid; *n*_*i*_ = number of synonymous codons for *i*_*t* *h*_ amino acid).

An RSCU value of 1.0 indicates neutral usage, while values < 0.6, 0.6–1.6, and >  1.6 denote underrepresented, typical, and overrepresented codons, respectively [12]. All pneumolysin sequences were analyzed using R (v4.1.1) with the *seqinr* package (v3.6–1).

### 2.4. Relative Dinucleotide Abundance Analysis

Relative dinucleotide abundance profiling was conducted for pneumolysin CDSs to identify compositional biases in nucleotide pair distributions. This metric evaluates the ratio of observed dinucleotide frequencies to expected frequencies (calculated from mononucleotide occurrences), where values below 0.78 indicate suppressed representation and values exceeding 1.23 denote overrepresentation of specific dinucleotides [13], and is computed using the formula:

(2) Pxy=fxyfxfy

(where fx and fy = frequencies of nucleotides X and Y; fxy = observed dinucleotide XY frequency).

All analyses were implemented in R v4.1.1.

### 2.5. Effective Number of Codons (ENC) Analysis

The ENC metric was applied to quantify codon usage bias in the pneumolysin gene, measuring its deviation from random synonymous codon selection independent of CDS length. ENC values range from 20 (extreme bias: exclusive use of one effective codon per amino acid) to 61 (no bias: uniform usage of all synonymous codons) [13]. For pneumolysin, values ≤ 45 indicate statistically significant codon preference [14].

ENC was computed using the formula [15]:

(3) ENC=2+9F2+1F3+5F4+3F6

where *F* represents the mean of *F*_*i*_ values (for amino acids with i‐fold degeneracy, *i* = 2, 3, 4, 6), calculated as follows:

(4) Fi=n∑j=1inj/n2−1n−1

### 2.6. Parity Rule 2 (PR2) Analysis

PR2 analysis was employed to assess mutational pressure versus natural selection forces shaping pneumolysin codon usage. Synonymous third‐position nucleotides were evaluated through AT‐skew [A3/(A3 + T3)] (*x*‐axis) and GC‐skew [G3/(G3 + C3)] (*y*‐axis). Central clustering at coordinates (0.5, 0.5)—where A3 = T3 and G3 = C3—indicates equilibrium between evolutionary forces. Deviation magnitude was quantified as distance from plot coordinates to this centroid [16].

### 2.7. ENC‐Plot Analysis

The ENC‐plot incorporates a theoretical curve representing the expected ENC‐GC3 relationship under complete absence of selection pressure. This curve is derived computationally using the established formula:

(5) ENCexpected=23+GC+29GC32+13−GC2

If observed ENC values fall significantly below this selection‐neutral baseline, it suggests that codon usage bias is influenced by evolutionary forces—particularly natural selection [13]. These analyses were conducted using R statistical software (v4.1.1).

### 2.8. Neutrality Plot Analysis

The neutrality plot is a graphical representation employed to evaluate the relative contributions of mutational pressure versus natural selection in shaping codon usage bias within the pneumolysin gene. This analysis plots the mean GC content at the first and second codon positions (GC12) along the vertical axis against the GC content at the synonymous third codon position (GC3) on the horizontal axis. Linear regression is applied to interpret the resulting data distribution. A regression slope approximating 1 indicates that mutational pressure predominantly dictates codon usage patterns for pneumolysin. Conversely, a slope approaching 0 signifies that natural selection exerts a stronger influence on the codon usage bias observed in pneumolysin [17].

### 2.9. Isoacceptor tRNA Pool Analysis

Distinct tRNA isoacceptors, which originate from diverse tRNA types, play an essential role in recognizing specific codons associated with amino acids. This precise recognition ensures the fidelity of amino acid incorporation into growing polypeptide chains during translation. Data on human tRNA gene abundance were obtained from the GtRNAdb database (https://gtrnadb.ucsc.edu) [18]. It is noteworthy that multiple isoacceptors can recognize a single codon—a phenomenon that varies across species and influences both gene expression levels and protein synthesis efficiency. Although pneumolysin is translated within the bacterial pathogen rather than human cells, this study specifically examines the abundance of human tRNA genes corresponding to the codons preferentially used in the pneumolysin gene. This approach helps elucidate potential mechanisms of host–pathogen interaction and highlights evolutionary adaptations that may facilitate immune evasion during infection.

### 2.10. Statistical Analysis

Quantitative data are expressed as mean values ± standard deviation (SD). All statistical procedures were conducted utilizing SPSS software (Release 22.0, IBM Corp.).

## 3. Results

### 3.1. Nucleotide Compositional Characteristics of the Pneumolysin Gene

Comprehensive nucleotide composition analysis revealed distinct patterns across the three codon positions of the pneumolysin gene (Table 1). The mean GC content at the first codon position (GC1) was the highest (51.58% ± 0.11%), followed by the GC content at the synonymous third position (GC3) (37.98% ± 0.26%). In contrast, the second codon position (GC2) exhibited the lowest GC content (36.44% ± 0.03%), reflecting marked preferential use of A/T‐ending codons. A pronounced predominance of A3s and T3s over G3s and C3s is observed (Table 1). Specifically, thymine (T3s) was the most abundant base at the third codon position (49.7% ± 0.19%), followed by adenine (A3s) (30.04% ± 0.26%), while guanine (G3s) and cytosine (C3s) were significantly less frequent (27.13% ± 0.32% and 19.85% ± 0.22%, respectively).

**TABLE 1:** Nucleotide compositional characteristics of the pneumolysin gene.

	GC1	GC2	GC3	T3s	C3s	A3s	G3s
Pneumolysin	51.58 ± 0.11	36.44 ± 0.03	37.98 ± 0.26	49.7 ± 0.19	19.85 ± 0.22	30.04 ± 0.26	27.13 ± 0.32

ENC value quantifies deviation from stochastic codon selection patterns. ENC values ≤ 45 demonstrate pronounced codon usage bias at genomic or gene‐specific levels. Analysis of pneumolysin yields an ENC of 50.28 ± 0.20 (Table S2), indicating limited codon preference.

### 3.2. Synonymous Codon Preference in Pneumolysin Based on RSCU Analysis

**FIGURE 1:**
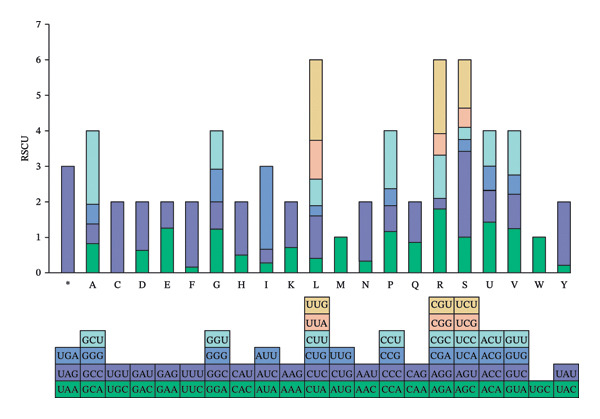
Relative synonymous codon usage (RSCU) analysis in the pneumolysin gene. The horizontal axis represents 20 amino acids and their corresponding synonymous codons. The vertical axis denotes the RSCU value of each codon, which reflects the usage bias of synonymous codons: RSCU > 1.6 indicates an overrepresented codon, 0.6 < RSCU < 1.6 indicates a typically used codon, and RSCU < 0.6 indicates an underrepresented codon. This plot intuitively shows the preference of the pneumolysin gene for A/U‐ending codons, with most overrepresented codons (RSCU > 1.6) terminating in A or U nucleotides.

RSCU analysis of the pneumolysin gene reveals preferential usage of 28 synonymous codons (RSCU > 1), with 22 containing 3’‐terminal A/U nucleotides. Eleven codons are particularly overrepresented (RSCU > 1.6): AGA, AAU, AGU, AUU, CCU, CGU, GCU, UAU, UGU, UUU, and UUG (Figure 1; Table 2). Conversely, 18 codons are underrepresented (RSCU < 0.6), 14 of which terminate in G/C. Both nucleotide composition analyses and RSCU values confirm a systematic bias toward A/U‐ending codons in pneumolysin. These findings indicate that synonymous codon selection in this gene is principally governed by third‐position nucleotide identity.

**TABLE 2:** Preferred codon count in the pneumolysin gene.

	Pneumolysin
Number of preferred codons (RSCU > 1)	28
Number of preferred codons ending in G/C	6
Number of overrepresented codons (RSCU > 1.6)	11

### 3.3. Dinucleotide Composition Effects on Pneumolysin Codon Selection

**FIGURE 2:**
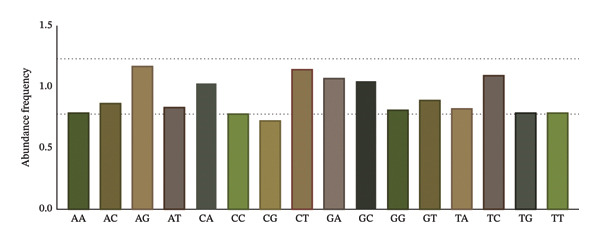
Dinucleotide frequency analysis in the pneumolysin gene. Dashed lines denote significance thresholds (overrepresented: Pxy > 1.23; underrepresented: Pxy < 0.78). This plot intuitively reflects the compositional bias of dinucleotides in the pneumolysin gene, with the CpG dinucleotide showing the most significant underrepresentation (Pxy < 0.78).

Analysis reveals substantial deviation of dinucleotide frequencies from expected values (Pxy ≠ 1.0) in pneumolysin. Notably, CpG dinucleotides exhibit significant depletion (observed‐to‐expected ratio < 0.78) as shown in Figure 2.

To assess dinucleotide effects on codon selection, underrepresented dinucleotides within disfavored codons were analyzed. Six of eight CpG‐containing codons (CCG, CGG, GCG, UCG, ACG, and CGA) exhibited RSCU values < 1.0, indicating systematic avoidance of CpG dinucleotides. The patterns observed in trinucleotide usage are consistent with these findings, as indicated by the RSCU data.

### 3.4. Factors Influencing the Codon Usage Pattern of the Pneumolysin Gene

The PR2 analysis revealed that the codons of the pneumolysin gene exhibit significant compositional bias. Most coordinate points are concentrated in the third quadrant, where G3/(G3 + C3) < 0.5 and A3/(A3 + T3) < 0.5, implying that strong mutational pressure or natural selection is acting on the gene (Figure 3(a)). Further supporting this, the ENC‐plot showed that most CDSs fall below the theoretical curve, demonstrating the stronger influence of natural selection on codon optimization (Figure 3(b)). Neutrality plot analysis revealed a slope of −0.0796; as this value approaches zero, it strongly indicates that natural selection, rather than mutational pressure, is the dominant force shaping codon usage bias (Figure 3(c)). Thus, while both mutational pressure and natural selection influence the codon usage patterns of the pneumolysin gene, natural selection plays the dominant role.

FIGURE 3Analysis of evolutionary forces shaping codon usage in the pneumolysin gene. (a) PR2 plot analysis: Each point represents a pneumolysin coding sequence. *X*‐Axis: GC‐skew [G3/(G3 + C3)]; *Y*‐Axis: AT‐skew [A3/(A3 + T3)]. The point (0.5, 0.5) indicates nucleotide usage balance (A3 = T3, G3 = C3); deviation from this center reflects the influence of mutational pressure or natural selection. (b) ENC‐Plot Analysis: Each point represents a pneumolysin coding sequence. *X*‐Axis: GC content at the third codon position (GC3); *Y*‐Axis: effective number of codons (ENC). The solid curve represents the expected ENC under no selection pressure; most points falling below this curve indicate that natural selection, rather than mutational pressure, shapes the codon usage bias. (c) Neutrality Plot Analysis: Each point represents a pneumolysin coding sequence. *X*‐Axis: GC3; *Y*‐Axis: average GC content at the first and second codon positions (GC12). The linear regression line (*y* = −0.0796x + 40.987, slope = −0.0796) is close to zero, further demonstrating that natural selection is the dominant evolutionary force governing codon usage in the pneumolysin gene.

**Figure:**
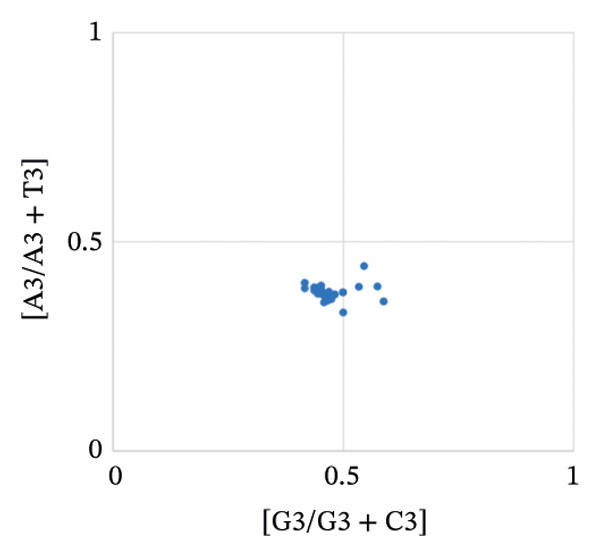
(a)

**Figure:**
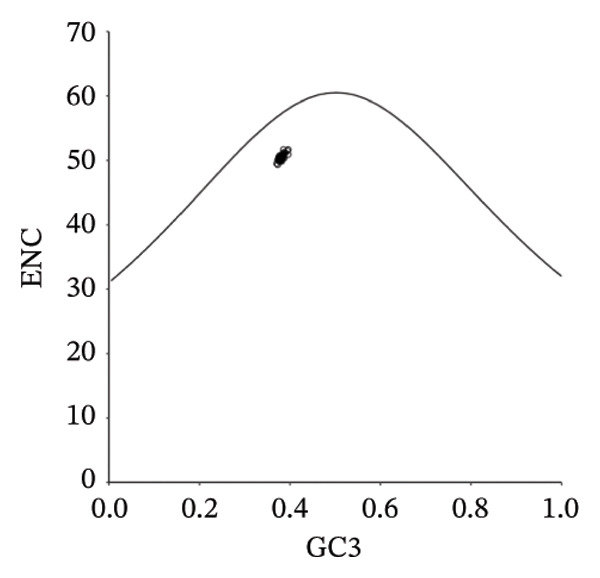
(b)

**Figure:**
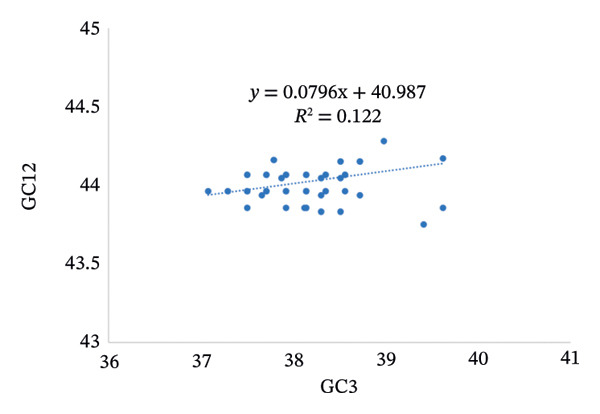
(c)

### 3.5. Abundance of Human tRNA Genes Complementary to the Frequent Codons in Pneumolysin

To explore the potential adaptation of pneumolysin to the host translational environment, its codon usage pattern was compared with the abundance of tRNA genes in *Homo sapiens*. Specifically, we aligned the RSCU values of pneumolysin with the genomic tRNA gene repertoire of humans. Notably, for multicodon amino acids, the codons exhibiting the highest RSCU values in pneumolysin (e.g., for Ala, Pro, Arg, Lys, Gln, and Ile) corresponded to codons decoded by the most prevalent human tRNA isoacceptors; and suboptimal pairing was observed for Gly, Thr, and Val (Table 3). Although pneumolysin is synthesized by the bacterial translational machinery, the observed overlap between its preferred codons and the abundant tRNA pool of the host may suggest an evolutionary strategy to minimize immune activation. It is plausible that this adaptation could potentially reduce the immunostimulatory capacity of bacterial mRNA released into the host environment upon bacterial lysis, thereby possibly aiding immune evasion and supporting the establishment of infection.

**TABLE 3:** Abundance of human tRNA genes complementary to the most frequent codons in pneumolysin.

Amino acid	Most preferred codons in pneumolysin	tRNA isotypes in human cells	Total count
Ala (A)	GCU	AGC (22), GGC (0), CGC (4), UGC (8)	34
Gly (G)	GGA	ACC (0), GCC (14), CCC (5), UCC (9)	28
Pro (P)	CCU	AGG (9), GGG (0), CGG (4), UGG (7)	20
Thr (T)	ACA	AGU (9), GGU (0), CGU (5), UGU (6)	20
Val (V)	GUU	AAC (9), GAC (0), CAC (11), UAC (5)	25
Ser (S)	AGU	AGA (9), GGA (0), CGA (4), UGA (4), ACU (0), GCU (8)	25
Arg (R)	CGU	ACG (7), GCG (0), CCG (4), UCG (6), CCU (5), UCU (6)	28
Leu (L)	UUG	AAG (9), GAG (0), CAG (9), UAG (3), CAA (6), UAA (4)	31
Phe (F)	UUU	AAA (0), GAA (10)	10
Asn (N)	AAU	AUU (0), GUU (20)	20
Lys (K)	AAG	CUU (15), UUU (12)	27
Asp (D)	GAU	AUC (0), GUC (13)	13
Glu (E)	GAA	CUC (8), UUC (7)	15
His (H)	CAU	AUG (0), GUG (10)	10
Gln (Q)	CAG	CUG (13), UUG (6)	19
Ile (I)	AUU	AAU (14), GAU (3), UAU (5)	22
Tyr (Y)	UAU	AUA (0), GUA (13)	13
Cys (C)	UGU	ACA (0), GCA (29)	29
Trp (W)	UGG	CCA (7)	7
Met (M)	AUG	CAU (9/10)	19

## 4. Discussion

The expression efficiency of *S. pneumoniae* pneumolysin, a key CDC virulence factor, is strongly influenced by synonymous codon usage bias in its encoding gene. Although silent at the amino acid level, this bias significantly affects translational efficiency, cotranslational folding, and functional protein yield [8, 9]. Our comprehensive computational analysis indicates that pneumolysin’s codon usage is not only influenced by mutational pressure but also predominantly shaped by natural selection, fine‐tuning its expression to maintain toxin function and facilitate host adaptation.

### 4.1. Codon Usage Patterns and Immune Evasion Strategy

We elucidate intricate codon usage patterns in pneumolysin, characterized by a pronounced nucleotide bias: depressed GC3 content (< 39%), strong preference for A/U‐ending codons (22 of 28 preferred codons exhibit RSCU > 1), and underrepresentation of predominantly G/C‐rich codons (14 of 18 underrepresented codons have RSCU < 0.6). Additionally, we observed marked depletion of CpG dinucleotides. We propose this represents a finely tuned adaptation to circumvent host defenses. Specifically, CpG depletion is salient because unmethylated CpG motifs act as potent ligands for Toll‐like receptor 9 (TLR9), triggering pro‐inflammatory responses [19, 20]. The systematic avoidance of CpG‐rich codons inherent in the observed bias likely constitutes an immune‐evasive tactic, reducing immunostimulatory signatures released during bacterial lysis and pneumolysin liberation [21]. This mechanism aligns with findings showing that CpG is the most underavoided dinucleotide in other bacterial protein toxins [22]. Furthermore, our analysis uncovered an additional layer of potential host adaptation: The codon usage pattern of pneumolysin shows significant compatibility with the tRNA gene pool of its human host (*Homo sapiens*). While pneumolysin is translated within the bacterial cytoplasm, this observed compatibility is unlikely to be coincidental. We propose that this represents a sophisticated evolutionary strategy to minimize the immunostimulatory potential of bacterial mRNA released upon lysis. By favoring codons that are efficiently decoded by the host’s abundant tRNAs, the bacterium may reduce the persistence and alter the immunogenic profile of its genetic material within the host environment, thereby facilitating immune evasion and supporting the establishment of infection [23].

### 4.2. Balancing Expression and Folding

The observed moderate codon bias in pneumolysin (ENC = 50.28 ± 0.20) presents a paradox: Despite pneumolysin’s critical role in virulence via host cell lysis and immune modulation, strong codon bias favoring maximal translational efficiency is absent. This paradox is resolved by considering the detrimental effects synonymous substitutions can have. Introducing optimized high‐frequency codons can disrupt protein folding pathways, leading to misfolding, aggregation, and degradation [24]. We therefore propose that extreme codon optimization might compromise translational fidelity and folding kinetics for this large (53 kDa) pore‐forming toxin [6]. Instead, the observed moderate bias likely represents a sophisticated evolutionary balance. It strategically ensures sufficiently high expression levels for pathogenicity while enabling the precise temporal control and stepwise progression of cotranslational folding essential for the nascent polypeptide chain. This delicate equilibrium between expression level and folding precision is absolutely critical for subsequent processes—proper monomer folding, stable oligomerization, and functional pore formation—ultimately maintaining the functional integrity paramount for virulence.

Furthermore, the weak codon usage bias (ENC > 45) observed in the pneumolysin gene may represent an adaptive evolutionary strategy. As an obligate pathogen primarily infecting humans, *S. pneumoniae* must adapt to diverse host microenvironments (e.g., nasopharynx, lungs, and blood). While strong codon bias optimizes translational efficiency under stable conditions, it may constrain regulatory flexibility [7]. Weak bias preserves a broader synonymous codon repertoire, enabling fine‐tuned control of translation rates and protein yield in response to fluctuating stresses encountered during infection [25]. Therefore, the weak codon bias in pneumolysin may reflect optimization not for maximal translational speed, but for regulatability, ensuring appropriate toxin production across distinct physiological contexts throughout the infection cycle. This adaptability is likely critical for a virulence factor involved in both local tissue damage and systemic immune modulation.

### 4.3. The Dominant Role of Natural Selection in Shaping Codon Usage

A central question in evolutionary genomics is whether codon usage bias arises from neutral mutational processes or is actively shaped by natural selection. A systematic analysis of 125 bacterial protein toxin genes from 49 pathogenic species by Sharma et al. [22] provides a critical comparative framework. Their study demonstrated that, despite diverse mechanisms of action across toxin families, codon usage exhibits several convergent features: universal AT enrichment, distribution of data points below the expected curve in GC3s‐ENC plots, and near‐zero regression slopes in neutrality plots. These signatures collectively indicate that natural selection is the dominant force shaping codon usage across bacterial protein toxins. The results of our pneumolysin analysis are highly consistent with this broader toxin paradigm: ENC‐plot positions below the neutral curve and a near‐zero neutrality plot slope. This concordance is not coincidental; rather, it suggests that although pneumolysin belongs to the CDC family, its codon usage evolution follows the general rules established for bacterial protein toxins—selective pressure has overridden the baseline influence of genomic mutational bias. Notably, Sharma et al. also reported subtle variations among toxin classes: Ribosome‐inactivating proteins (RIPs) exhibited relatively higher GC3 content and shared only 50% of their preferred codons with the parental genome. This heterogeneity implies that, within the overarching framework of selection‐driven evolution, individual toxin genes may adopt distinct adaptive trajectories.

### 4.4. Dual Applications: Expression Optimization and Vaccine Design

Pneumolysin is a major virulence factor of *S. pneumoniae* and a prime target for therapeutic interventions and vaccine development. Therefore, understanding its codon usage bias is not only of evolutionary interest but also has immediate practical implications. The detailed characterization of pneumolysin’s codon preferences and the evolutionary forces shaping them provides a rational foundation for two complementary applications. Including this translational perspective in the Discussion is essential to demonstrate how our findings can be directly leveraged to address key challenges in pneumococcal disease research and control.

Recombinant Protein Production: Knowledge of preferred codons enables engineering heterologous expression systems (e.g., *Escherichia coli*, yeast, and mammalian cells) to match pneumolysin’s codon preferences or supply tRNAs for its rare codons (particularly underrepresented G/C‐ending codons). This optimization is vital to produce sufficient quantities of correctly folded, functional pneumolysin required for structural studies (X‐ray crystallography and cryo‐EM), antibody development, and functional assays, overcoming bottlenecks caused by codon mismatch [8, 26].

Vaccine Development: Conversely, understanding that natural selection optimized pneumolysin codons for high expression in humans enables a deliberate codon deoptimization (CD) strategy. By synthetically replacing preferred codons (especially those matching abundant human tRNAs) with synonymous unpreferred codons (e.g., low‐RSCU G/C‐ending codons recognized by rare human tRNAs), pneumolysin’s translational efficiency can be drastically reduced without altering its amino acid sequence. Introducing such a CD pneumolysin gene into *S. pneumoniae* vaccine strains could generate live‐attenuated bacteria capable of colonization and robust immune priming, while being severely hampered in pneumolysin production and thus virulence, significantly enhancing safety. The RSCU and tRNA matching data provide specific targets for both optimizing heterologous expression and rationally designing translational attenuation for vaccines [10, 27, 28].

## 5. Conclusion

This study conducted a comprehensive analysis of the codon usage bias in the gene encoding *S. pneumoniae* pneumolysin, a major virulence factor. Results revealed a significant preference for A/U‐ending codons, strong suppression of CpG dinucleotides, and a moderate overall bias (ENC = 50.28). Analytical approaches including RSCU, PR2, ENC‐plot, and neutrality plot demonstrated that natural selection, not mutational pressure, is the dominant evolutionary force shaping this codon usage pattern. Strikingly, pneumolysin’s preferred codons show a significant correlation with the abundant tRNA gene pool of its human host, suggesting an immunoevasive strategy to reduce the immunostimulatory impact of bacterial mRNA released during lysis. This reflects an evolutionary balance between ensuring efficient toxin expression and folding in the bacterium and evading host immunity. The findings provide a basis for optimizing recombinant pneumolysin production and inform codon deoptimization strategies for developing live‐attenuated vaccines.

## Author Contributions

Weifeng Shen made a contribution to the research conception. Xiaochun Tan and Hui Zhou collected information from the database. Xiaochun Tan and Jian Jiang analyzed and explained the data. Xiaochun Tan and Yu Lu are the main contributors to the manuscript. Junyan Lu helped with the analysis through constructive discussion.

## Funding

The study was funded by the Jiaxing Key Discipline of Medicine—Clinical Diagnostics (Grant No. 2023‐ZC‐002), the Jiaxing Key Laboratory of Clinical Laboratory Diagnostics and Translational Research (Grant No. 2023‐lcjyzdyzh), the “Venus” Talent Training Program of the Jiaxing First Hospital (Grant No. 2025‐QXM‐008), and the Zhejiang Provincial Medical and Health Technology Program (Grant No. 2025KY354).

## Disclosure

All the authors read and approved the final manuscript.

## Ethics Statement

The authors have nothing to report.

## Conflicts of Interest

The authors declare no conflicts of interest.

## Supporting Information

Table S1. Pneumolysin gene accession numbers used in this study.

Table S2. Effective Number of Codons (ENC) values of the pneumolysin gene.

## Supporting information


**Supporting Information** Additional supporting information can be found online in the Supporting Information section.

## Data Availability

The supporting data for the findings presented in this study are accessible through the GenBank database at https://www.ncbi.nlm.nih.gov/Genbank. Reference numbers are provided in the Table S1 accompanying this article.
