# Supplementary material for: Analysis of Codon Usage Bias in the Streptococcus pneumoniae Pneumolysin Gene
Source: Can J Infect Dis Med Microbiol. 2026 Apr 5;2026:2707639. doi: 10.1155/cjid/2707639 (PMC13051798; doi:10.1155/cjid/2707639)
Supplement: Supplementary file 1 — Supporting Information Additional supporting information can be found online in the Supporting Information section. [file CJID-2026-2707639-s001.docx]

Table S1. Pneumolysin gene accession numbers used in this study.

| Accession numbers |
| --- |
| MK286385.1 |
| MK286384.1 |
| MK286383.1 |
| MK286382.1 |
| MK286381.1 |
| MK286380.1 |
| MK286379.1 |
| GU968403.1 |
| GU968404.1 |
| GU968405.1 |
| GU968406.1 |
| GU968407.1 |
| GU968408.1 |
| GU968409.1 |
| GU968410.1 |
| GU968411.1 |
| HQ711618.1 |
| KP110598.1 |
| KP110599.1 |
| KP110600.1 |
| KP110601.1 |
| KP110602.1 |
| KP110603.1 |
| KP110604.1 |
| KP110605.1 |
| KP110606.1 |
| KP110607.1 |
| KP110608.1 |
| KP110609.1 |
| KP110610.1 |
| KP110611.1 |
| KP110612.1 |
| KP110613.1 |
| KP110614.1 |
| KP110615.1 |
| KP110616.1 |
| KP110617.1 |
| KP110618.1 |
| KP110619.1 |
| KP110620.1 |
| KP110621.1 |
| KP110622.1 |
| KP110623.1 |
| KP110624.1 |
| KP110625.1 |
| KP110626.1 |
| KP110627.1 |
| KP110628.1 |
| KP110629.1 |
| KP110630.1 |
| KP110631.1 |
| KP110632.1 |
| KP110633.1 |
| KP110634.1 |
| KP110635.1 |
| KP110636.1 |
| KP110637.1 |
| KP110638.1 |
| KP110639.1 |
| KP110640.1 |
| KP110641.1 |
| KP110642.1 |
| KP110643.1 |
| KP110644.1 |
| KP110645.1 |
| KP110646.1 |
| KP110647.1 |
| KP110648.1 |
| KP110649.1 |
| KP110650.1 |
| MK286378.1 |
| MK286377.1 |
| MK286376.1 |
| KP110770.1 |
| KP110769.1 |
| KP110768.1 |
| KP110767.1 |
| KP110766.1 |
| KP110765.1 |
| KP110764.1 |
| KP110763.1 |
| KP110762.1 |
| KP110761.1 |
| KP110760.1 |
| KP110759.1 |
| KP110758.1 |
| KP110757.1 |
| KP110756.1 |
| KP110755.1 |
| KP110754.1 |
| KP110753.1 |
| KP110752.1 |
| KP110751.1 |
| KP110750.1 |
| KP110749.1 |
| KP110748.1 |
| KP110747.1 |
| KP110746.1 |
| KP110745.1 |
| KP110744.1 |
| KP110743.1 |
| KP110742.1 |
| KP110741.1 |
| KP110740.1 |
| KP110739.1 |
| KP110738.1 |
| KP110737.1 |
| KP110736.1 |
| KP110735.1 |
| KP110734.1 |
| KP110733.1 |
| KP110732.1 |
| KP110731.1 |
| KP110730.1 |
| KP110729.1 |
| KP110728.1 |
| KP110727.1 |
| KP110726.1 |
| KP110725.1 |
| KP110724.1 |
| KP110723.1 |
| KP110722.1 |
| KP110721.1 |
| KP110720.1 |
| KP110719.1 |
| KP110718.1 |
| KP110717.1 |
| KP110716.1 |
| KP110715.1 |
| KP110714.1 |
| KP110713.1 |
| KP110712.1 |
| KP110711.1 |
| KP110710.1 |
| KP110709.1 |
| KP110708.1 |
| KP110707.1 |
| KP110706.1 |
| KP110705.1 |
| KP110704.1 |
| KP110703.1 |
| KP110702.1 |
| KP110701.1 |
| KP110700.1 |
| KP110699.1 |
| KP110698.1 |
| KP110697.1 |
| KP110696.1 |
| KP110695.1 |
| KP110694.1 |
| KP110693.1 |
| KP110692.1 |
| KP110691.1 |
| KP110690.1 |
| KP110689.1 |
| KP110688.1 |
| KP110687.1 |
| KP110686.1 |
| KP110685.1 |
| KP110684.1 |
| KP110683.1 |
| KP110682.1 |
| KP110681.1 |
| KP110680.1 |
| KP110679.1 |
| KP110678.1 |
| KP110677.1 |
| KP110676.1 |
| KP110675.1 |
| KP110674.1 |
| KP110673.1 |
| KP110672.1 |
| KP110671.1 |
| KP110670.1 |
| KP110669.1 |
| KP110668.1 |
| KP110667.1 |
| KP110666.1 |
| KP110651.1 |
| KP110652.1 |
| KP110653.1 |
| KP110654.1 |
| KP110655.1 |
| KP110656.1 |
| KP110657.1 |
| KP110658.1 |
| KP110659.1 |
| KP110660.1 |
| KP110661.1 |
| KP110662.1 |
| KP110663.1 |
| KP110664.1 |
| KP110665.1 |
| GU968395.1 |
| GU968394.1 |
| GU968393.1 |
| GU968392.1 |
| GU968391.1 |
| GU968390.1 |
| GU968389.1 |
| GU968388.1 |
| GU968387.1 |
| GU968386.1 |
| GU968385.1 |
| GU968384.1 |
| GU968383.1 |
| GU968382.1 |
| GU968381.1 |
| GU968380.1 |
| GU968379.1 |
| GU968378.1 |
| GU968377.1 |
| GU968376.1 |
| GU968375.1 |
| GU968374.1 |
| GU968373.1 |
| GU968372.1 |
| GU968371.1 |
| GU968370.1 |
| GU968369.1 |
| GU968368.1 |
| GU968367.1 |
| GU968366.1 |
| GU968365.1 |
| GU968364.1 |
| GU968363.1 |
| GU968362.1 |
| GU968361.1 |
| GU968360.1 |
| GU968359.1 |
| GU968358.1 |
| GU968357.1 |
| GU968356.1 |
| GU968355.1 |
| GU968354.1 |
| GU968353.1 |
| GU968352.1 |
| GU968351.1 |
| GU968350.1 |
| GU968349.1 |
| GU968348.1 |
| GU968347.1 |
| GU968346.1 |
| GU968345.1 |
| GU968344.1 |
| EF413939.1 |
| EF413940.1 |
| EF413941.1 |
| EF413942.1 |
| EF413943.1 |
| EF413944.1 |
| EF413945.1 |
| EF413946.1 |
| EF413947.1 |
| EF413949.1 |
| EF413950.1 |
| EF413951.1 |
| EF413952.1 |
| EF413953.1 |
| EF413954.1 |
| EF413955.1 |
| EF413956.1 |
| EF413957.1 |
| EF413958.1 |
| EF413959.1 |
| EF413960.1 |
| GU968217.1 |
| GU968218.1 |
| GU968219.1 |
| GU968220.1 |
| GU968221.1 |
| GU968222.1 |
| GU968223.1 |
| GU968224.1 |
| GU968225.1 |
| GU968226.1 |
| GU968227.1 |
| GU968228.1 |
| GU968229.1 |
| GU968343.1 |
| GU968342.1 |
| GU968341.1 |
| GU968340.1 |
| GU968339.1 |
| GU968338.1 |
| GU968337.1 |
| GU968336.1 |
| GU968335.1 |
| GU968334.1 |
| GU968333.1 |
| GU968332.1 |
| GU968331.1 |
| GU968330.1 |
| GU968329.1 |
| GU968328.1 |
| GU968327.1 |
| GU968326.1 |
| GU968325.1 |
| GU968324.1 |
| GU968323.1 |
| GU968322.1 |
| GU968321.1 |
| GU968320.1 |
| GU968319.1 |
| GU968318.1 |
| GU968317.1 |
| GU968316.1 |
| GU968315.1 |
| GU968314.1 |
| GU968313.1 |
| GU968312.1 |
| GU968311.1 |
| GU968310.1 |
| GU968309.1 |
| GU968308.1 |
| GU968307.1 |
| GU968306.1 |
| GU968305.1 |
| GU968304.1 |
| GU968303.1 |
| GU968302.1 |
| GU968301.1 |
| GU968300.1 |
| GU968299.1 |
| GU968298.1 |
| GU968297.1 |
| GU968296.1 |
| GU968295.1 |
| GU968294.1 |
| GU968293.1 |
| GU968292.1 |
| GU968291.1 |
| GU968290.1 |
| GU968289.1 |
| GU968288.1 |
| GU968287.1 |
| GU968286.1 |
| GU968285.1 |
| GU968284.1 |
| GU968283.1 |
| GU968282.1 |
| GU968281.1 |
| GU968280.1 |
| GU968279.1 |
| GU968278.1 |
| GU968277.1 |
| GU968276.1 |
| GU968275.1 |
| GU968274.1 |
| GU968273.1 |
| GU968272.1 |
| GU968271.1 |
| GU968270.1 |
| GU968269.1 |
| GU968268.1 |
| GU968267.1 |
| GU968266.1 |
| GU968265.1 |
| GU968264.1 |
| GU968263.1 |
| GU968262.1 |
| GU968261.1 |
| GU968260.1 |
| GU968259.1 |
| GU968258.1 |
| GU968257.1 |
| GU968256.1 |
| GU968255.1 |
| GU968254.1 |
| GU968253.1 |
| GU968252.1 |
| GU968251.1 |
| GU968250.1 |
| GU968249.1 |
| GU968248.1 |
| GU968247.1 |
| GU968246.1 |
| GU968245.1 |
| GU968244.1 |
| GU968243.1 |
| GU968242.1 |
| GU968241.1 |
| GU968240.1 |
| GU968239.1 |
| GU968238.1 |
| GU968237.1 |
| GU968236.1 |
| GU968235.1 |
| GU968234.1 |
| GU968233.1 |
| GU968232.1 |
| GU968231.1 |
| GU968230.1 |
| EF413938.1 |
| EF413937.1 |
| EF413936.1 |
| EF413935.1 |
| EF413934.1 |
| EF413933.1 |
| EF413932.1 |
| EF413931.1 |
| EF413930.1 |
| EF413929.1 |
| EF413928.1 |
| EF413927.1 |
| EF413926.1 |
| EF413925.1 |
| EF413924.1 |
| EF413923.1 |
| DQ251181.1 |
| DQ251180.1 |
| DQ251179.1 |
| DQ251178.1 |
| DQ251177.1 |
| KP982898.1 |
| EF368014.1 |
| A03623.1 |
| FJ440116.1 |
| FJ440115.1 |
| FJ440114.1 |

Table S2. Effective Number of Codons (ENC) values of the pneumolysin gene.

|  | ENC |
| --- | --- |
| Pneumolysin | 50.28±0.20 |
